# Molecular Variations in Glycoprotein B of Asian Human Cytomegalovirus: Potential Impact on Virus Entry and Immune Evasion in Ocular Diseases

**DOI:** 10.1002/jmv.70786

**Published:** 2026-01-07

**Authors:** Tantri Lestari, Nobuyo Yawata, Gabriel Gonzalez, Hiroko Miyadera, Daisuke Motooka, Yuko Imamura, Hiroya Oki, Yasuo Mori, Mariko Shirane, Seik‐Soon Khor, Yosuke Omae, Mihoko Shimada, Dyah Ayu Windy, Satoko Nakano, Hiroki Tsutsui, Shiori Kuramoto, Chihiro Fukui, Riku Nakamura, Satoshi Yamana, Toshikatsu Kaburaki, Hisashi Mashimo, Hiroshi Takase, Ryoji Yanai, Eiichi Hasegawa, Kensuke Shibata, Makoto Yawata, Katsushi Tokunaga, Nobuyuki Ohguro, Koh‐Hei Sonoda

**Affiliations:** ^1^ Department of Ophthalmology Kyushu University Fukuoka Japan; ^2^ Department of Ocular Pathology and Imaging Science Kyushu University Fukuoka Japan; ^3^ Singapore Eye Research Institute Singapore; ^4^ Ophthalmology and Visual Sciences Academic Clinical Program Duke‐NUS Medical School Singapore; ^5^ Institute for Vaccine Research and Development Hokkaido University Sapporo Japan; ^6^ Department of Medical Genetics, Institute of Medicine University of Tsukuba Tsukuba Japan; ^7^ Research, Institute for Microbial Diseases The University of Osaka Osaka Japan; ^8^ Department of Medicine and Biosystemic Science Kyushu University Fukuoka Japan; ^9^ Genome Medical Science Project, National Institute of Global Health and Medicine Japan Institute for Health Security Tokyo Japan; ^10^ Singapore Centre for Environmental Life Sciences Engineering Nanyang Technological University Singapore; ^11^ Department of Ophthalmology Hasanuddin University Indonesia Makassar Indonesia; ^12^ Department of Ophthalmology Oita University Faculty of Medicine Oita Japan; ^13^ Department of Ophthalmology Jichi Medical University Shimono Tochigi Japan; ^14^ Japan Public Health Organization Osaka Hospital Osaka Japan; ^15^ Miyata Eye Hospital Tokyo Clinic Tokyo Japan; ^16^ Department of Ophthalmology Tokushima University Tokushima Japan; ^17^ Department of Visual Regeneration, Graduate School of Medical Sciences Kyushu University Fukuoka Japan; ^18^ Department of Molecular Immunology, Research Institute for Microbial Diseases Osaka University Osaka Japan; ^19^ Department of Molecular and Cellular Physiology, Graduate School of Medicine Yamaguchi University Ube Yamaguchi Japan; ^20^ Department of Pediatrics, Yong Loo Lin School of Medicine National University of Singapore Singapore; ^21^ Immunology Programme, Life Sciences Institute National University of Singapore Singapore; ^22^ NUSMED Immunology Translational Research Programme National University of Singapore Singapore

**Keywords:** CMV anterior uveitis, CMV retinitis, CMV viremia, Furin Cleavage Site, glycoprotein B, human cytomegalovirus, UL55

## Abstract

Human cytomegalovirus (HCMV)‐associated ocular diseases have gained increasing attention due to a recent rise in cases diagnosed in Asia. A glycoprotein encoded by the virus UL55 gene, glycoprotein B (gB), is essential for viral entry and a primary target for naturally‐produced antibodies and vaccine development. gB is classified into five genotypes (gB1–gB5) based on polymorphisms surrounding the furin cleavage site. This study analyzed the UL55 gene in 62 blood and ocular specimens of Japanese patients with CMV viremia and CMV‐associated ocular diseases. Distinct gB genotype distributions were found between sample types (*p* = 0.008): gB2 was the most prevalent genotype in blood samples (41%, 11/27), while gB3 (43%, 15/35) and gB1 (37%, 13/35) predominated in ocular fluids. Viral loads were significantly higher in gB1 and gB3‐positive samples compared with gB2 (*p* = 0.016). A shared gB1/gB3‐specific peptide (aa 190–204; SRVIAGTVFVAYHRD), distinct from that of gB2, exhibited reduced HLA class II binding. In addition, a K518R substitution was identified in 80% of gB1 and gB3 variants in our cohort and other Asian‐derived GenBank entries, but only 3% of European origin strains. This substitution was significantly enriched in ocular fluids from patients with CMV ocular infection (71%, 17/24), compared with blood from patients with CMV viremia (32%, 8/25) (*p* = 0.01). The predicted structural modeling infers that this substitution is located in the core of gB Domain III, and potentially increase the local molecular stability in this region. Evolutionary analyses indicated positive selective pressure at this site, implying the biological significance. These findings infer that genetic variations enriched in ocular fluids and Asian‐derived HCMV strains, may contribute to ocular pathogenesis through influencing on viral entry and reduced immune recognition.

## 1 Introduction

Human cytomegalovirus (HCMV) is a ubiquitous beta‐herpesvirus that typically causes asymptomatic or mild infection in immunocompetent individuals [1]. However, in immunocompromised hosts, such as individuals with acquired immunodeficiency syndrome or those undergoing hematopoietic stem cell transplantation, HCMV can lead to severe complications, including CMV retinitis [1]. Over the past decade, the spectrum of HCMV‐associated ocular diseases has broadened with the emergence of CMV chronic retinal necrosis (CRN) and CMV anterior uveitis. CRN is usually seen in individuals with moderate immunosuppression, whereas CMV anterior uveitis is increasingly recognized in immunocompetent patients [2, 3, 4]. These ocular diseases are generally refractory to treatment, vision‐threatening, and lack curative therapies.

HCMV establishes a latent infection in circulating leukocytes and disseminates hematogenously to various tissues. CMV viremia is detected in approximately 50% of patients following hematopoietic stem cell transplantation; however, only 5%–11% develop CMV retinitis, highlighting a gap in our understanding of tissue‐specific disease development [5]. More enigmatic is the discovery of CMV anterior uveitis in immunocompetent individuals. Notably, numerous cases have been reported in East Asian populations, while it is infrequent in Caucasian populations suggesting potential ethnic or regional susceptibility [6, 7, 8, 9, 10, 11].

HCMV possesses a large DNA genome (~ 236 kb) encoding over 170 open reading frames. Its genetic complexity enables infection of diverse host cell types through varied mechanisms for cellular entry, replication, and immune evasion [12, 13]. Among the viral proteins, glycoprotein B (gB), encoded by UL55, is a key envelope protein facilitating viral entry by mediating attachment and fusion with host cell membranes [14]. In addition to this essential role in infection, gB is a dominant target for naturally produced neutralizing antibodies and is a major focus of HCMV vaccine development [15, 16]. Most individuals exhibit robust CD4⁺T cell responses against gB, underscoring its immunodominance [17, 18].

gB is classified into five genotypes (gB1–gB5) based on polymorphisms surrounding the furin cleavage site, a functionally critical region regulating the conformational transition of gB from prefusion to postfusion [19, 20]. These variations are supposed to influence viral tropism, transmissibility, and pathogenicity [21, 22]. The importance of polymorphisms in furin cleavage site has also been highlighted in other viral systems, such as SARS‐CoV‐2, where such variations facilitate enhanced cell‐to‐cell transmission [23].

Several studies have associated gB genotypes with clinical outcomes or tissue tropism, though findings are inconsistent [24, 25, 26]. To date, most studies on HCMV gB genotypes have relied on single type of clinical specimens derived from a single anatomical site. Therefore, whether the observed genotypic distribution reflects tissue‐specific tropism or regional predominance remain unsolved. In this study, we have investigated HCMV genotype distributions in peripheral blood and in the eye to identify eye‐specific determinants of HCMV. We also assessed the immunological properties and potential functional implications of gB polymorphisms. Furthermore, we compared our findings with Asian and European origin HCMVs in the GenBank database to explore regional viral features and their possible roles in disease pathogenesis.

## 2 Materials and Methods

### 2.1 Study Participants

This study enrolled 109 individuals diagnosed with CMV infection. Among them, 27 had CMV viremia, while 82 presented with CMV‐associated ocular diseases, including CMV retinitis (*n* = 24), anterior uveitis (*n* = 48), and CRN (*n* = 10). Participants were recruited between 2013 and 2024 from multiple medical institutions in Japan, including Kyushu University Hospital, the University of Tokyo Hospital, Japan Community Health Care Organization, Osaka Hospital, and Tokyo Medical and Dental University. Ocular CMV diseases were diagnosed via polymerase chain reaction (PCR) analysis of intraocular fluid samples. CMV viremia was diagnosed by CMV pp65 antigenemia. Virus copy numbers were also quantified as described before [27]. All patients with CMV viremia and CMV retinitis were immunocompromised, primarily due to treatment for hematological malignancies, with the exception of one patient with sarcoma and one with rheumatoid arthritis. Among those with hematological malignancies, 77% had undergone hematopoietic stem cell transplantation before developing CMV viremia and/or CMV retinitis. In contrast, all patients with CMV anterior uveitis were immunocompetent and were not receiving systemic immunosuppressive therapy; two had diabetes mellitus. Patients with CRN exhibited a mild to moderate degree of immunosuppression, including diabetes mellitus, use of immunosuppressants following liver transplantation, or localized immunosuppression from prior intravitreal triamcinolone acetonide injection.

The study adhered to the Declaration of Helsinki and received approval from the Institutional Review Board of Kyushu University Hospital. Written informed consent was obtained from all participants.

### 2.2 Specimen Collection and Viral DNA Extraction

Blood samples were collected from donors diagnosed with CMV viremia (*n* = 27) and CMV‐associated ocular diseases (*n* = 82). In patients with CMV‐associated ocular diseases, intraocular fluid samples (aqueous or vitreous humor) were obtained during routine diagnostic procedures. Viral DNA was extracted from 27 plasma and 35 intraocular fluid samples using the QIAamp MinElute Virus Spin Kit (Qiagen, Netherlands).

#### 2.2.1 Amplification and Sequencing of the UL55 Gene

**Figure 1:**
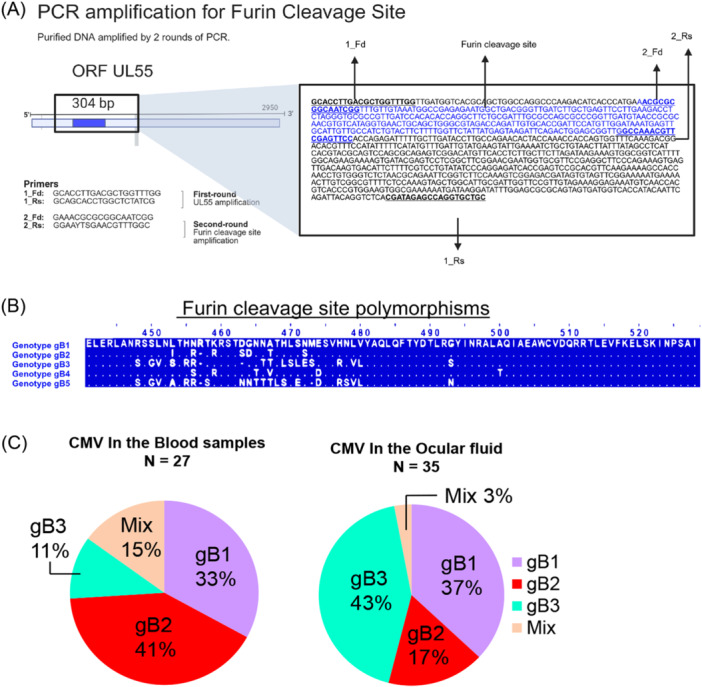
Distinct distribution of HCMV glycoprotein B genotypes in blood and intraocular fluids of CMV‐associated diseases. (A) Schematic of PCR strategy to amplify furin cleavage site in the UL55 region of HCMV. The first PCR reaction utilizes primers 1_Fd and 1_Rs to amplify a 974 bp region of the UL55 gene. The second PCR amplifies the furin cleavage site with primers 2_Fd and 2_Rs. (B) The amino acid sequences of the furin cleavage site across gB1–gB5 genotypes are shown with the Merlin sequence as reference (NC_006273). (C) Distribution of HCMV glycoprotein B (gB) genotypes in blood (*n* = 27) and ocular fluid samples (*n* = 35). The pie charts illustrate the significant differences in genotype distribution between the two compartments (*p* = 0.008, Fisher's exact test).

The UL55 gene, encoding gB, was amplified using two sets of primers targeting the furin‐cleavage site region (Figure 1A). In the first round of PCR, the primer pair F: 5′‐GCACCTTGACGCTGGTTTGG‐3′ and R: 5‐GCAGCACCTGGCTCTATCG‐3′ was used, as previously described [28]. PCR amplification was carried out with KOD FX polymerase (Toyobo, Osaka, Japan) under the following thermocycling conditions: initial denaturation at 94°C for 2 min; 35 cycles of denaturation at 98°C for 10 s, annealing at 55°C for 45 s, and extension at 68°C for 2 min; followed by a final extension at 68°C for 7 min. A second‐round PCR was performed using 1 µL of purified product from the first round as template. The nested primer pair used was F: 5′‐GAAACGCGCGGCAATCGG‐3 and R: 5′‐GGAAYTSGAACGTTTGGC‐3′, designed to amplify a 304 bp region encompassing the furin‐cleavage site [25]. Thermal cycling conditions were initial denaturation at 94°C for 2 min; 15 cycles of denaturation at 98°C for 10 s, annealing at 55°C for 40 s, and extension at 68°C for 50 s; followed by a final extension at 68°C for 7 min. Purified amplicons were subjected to Sanger sequencing. Resulting sequences were analyzed using BioEdit software to determine the HCMV gB genotypes based on polymorphisms within the furin‐cleavage site region (Figure 1B).

Presence of peptide sequences encoded in the gB gene was confirmed by direct sequencing of the amplicons using F: 5′‐ATAATCGTCGGGCATTAATTGC‐3′ and R: 5′‐ACGAGTCTACCAGAAGGTTT‐3′ under the following conditions: 35–45 cycles of denaturation at 98°C for 10 s, annealing at 56°C for 40 s, and extension at 68°C for 2 min, followed by a final extension at 68°C for 7 min.

#### 2.2.2 Multiplex SSP‐PCR for Glycoprotein B Genotyping

Following the initial PCR, multiplex sequence‐specific primer (SSP) PCR was performed to genotype the gB gene. The following primer sets were used:

FgB1: 5′‐ATGACCGCCACTTTCTTATC‐3′

FgB2: 5′‐TTCCGACTTTGGAAGACCCAA‐3′

FgB3: 5′‐TAGCTCCGGTGTGAACTCC‐3′

FgB4: 5′‐ACCATTCGTTCCGAAGCCGAGGAA‐3′

FgB5: 5′‐TACCCTATCGCTGGAGAAC‐3′

RgB: 5′‐GTTGATCCACACACCAGGC‐3′

These primers enabled the simultaneous detection of the five major HCMV gB genotypes and identification of mixed infections in a single sample, as previously described [26]. PCR amplification was carried out using the QIAGEN Multiplex PCR Kit (Qiagen, Cat. No. 206143) with the following thermal cycling conditions: initial denaturation at 95°C for 15 min; 15 cycles of 94°C for 30 s, 60°C for 90 s, and 72°C for 90 s; followed by a final extension at 72°C for 10 min.

#### 2.2.3 HLA Typing

Genomic DNA was extracted from 62 whole blood samples of patients with CMV‐associated ocular diseases using the QIAamp Blood Mini Kit (Qiagen). HLA typing for *HLA‐DRB1*, *DQB1*, and *DPB1* alleles was performed on patients with ocular CMV diseases, including CMV anterior uveitis (*n* = 38), CMV retinitis (*n* = 18), and CRN (*n* = 6). Genotyping was conducted using the AllType™ NGS kit (One Lambda, West Hills, CA, USA), following the manufacturer's instructions, as previously described [29]. HLA genotypes of patients with CMV viremia were obtained retrospectively from clinical records.

#### 2.2.4 Selection of Candidate Peptides and Binding Prediction

Fifteen‐mer peptides derived from the UL55 gene that elicited strong CD4⁺T cell responses and exhibited sequence variation across gB genotypes were selected based on a previous study [30]. Binding affinities of these peptides to HLA class II (HLA II) allotypes were predicted using NetMHCIIpan‐4.3 (https://services.healthtech.dtu.dk/services/ NetMHCIIpan‐4.3/) [31]. The output %Rank values were used to estimate binding strength, with lower %Rank values indicating stronger predicted affinity. Peptide–HLA II combinations with a %Rank above 5, indicating weak binding affinity, were excluded. Among the remaining pairs, those that exhibited more than a twofold difference in %Rank values between gB genotypes and HLA II allotypes present in our cohort were selected for further analysis.

#### 2.2.5 Measurement of Peptide Binding to HLA II

Peptide binding to HLA II molecules was evaluated by measuring the cell‐surface expression of HLA–peptide complexes, as previously described [32]. Briefly, stable cell lines expressing HLA II α subunits were generated by retroviral transduction of the NIH3T3 cell line (RIKEN Bioresource Center) using the pMXs‐puro vector and the packaging cell line PLAT‐E [33]. Double oligonucleotides encoding a signal sequence and the peptides were synthesized (Azenta Japan Corporation). Fusion constructs of HLA II β subunits and peptides were cloned into the pMXs‐IG, which contains an internal ribosome entry site (IRES) and a GFP reporter downstream of the HLA II β subunit–peptide fusion gene [34]. These constructs were transiently transfected into the α subunit‐expressing stable cell lines. Forty‐eight hours post‐transfection, cells were stained with an anti‐pan HLA II β monoclonal antibody (clone WR18; Cat# MCA477, Bio‐Rad Laboratories, Inc.) and phycoerythrin (PE)‐conjugated goat F(ab′)₂ anti‐mouse Ig (human adsorbed) and subjected to flow cytometric analysis using SA3800 cell analyzer (Sony Imaging Products & Solutions Inc.). The median fluorescence intensity (MFI) of cell‐surface HLA and cytoplasmic GFP was measured in GFP‐positive cells. The ratio of HLA to GFP was normalized to the ratio obtained for a negative control peptide (g15, a 15‐mer glycine peptide). This normalized value, termed the *g15 ratio*, served as an indicator of the surface expression of HLA–peptide complexes (Miyadera et al., manuscript in preparation). Data analysis was performed using FCS Express 6 software (version 6.06.0022; De Novo Software).

#### 2.2.6 Protein Structure Analysis

Amino acid sequences of gB were analyzed to predict structural and functional domains using PyMOL (Schrödinger, LLC). Three‐dimensional (3D) structural modeling was conducted based on the crystal structure of the extracellular domain of Human Cytomegalovirus gB (PDB ID: 5CXF), retrieved from the Protein Data Bank [16]. The Mutagenesis Wizard in PyMOL was utilized to assess the potential structural and functional impact of amino acid substitutions. Site‐directed mutagenesis was performed, and the rotamer conformation predicted to have the highest number of potential interactions and lowest strain energy was selected for detailed analysis. Interatomic distances were measured using the Measurement Wizard in PyMOL, with a focus on changes in hydrogen bonding and steric interactions. Substitutions were considered structurally significant when the distance between interacting atoms was less than 4 Å [35].

#### 2.2.7 Analysis for Selective Pressure on UL55

To identify sites under potential positive selection within the UL55 gene, selective pressure analysis was performed using MrBayes 3.2 software [36]. The Markov Chain Monte Carlo (MCMC) method was employed to estimate the posterior distribution of model parameters. Bayesian posterior probability analysis was conducted, with the ratio of non‐synonymous to synonymous substitutions (*ω* = *d*_N_/*d*_S_) used to assess selective pressure at individual codons [37]. Codons with a positive selection posterior probability (pr +) ≧ 25% were considered to be under positive selection. The Bayesian analyses were run with chain lengths assuring effective sample size (ESS) ≧ 100 for all model parameters and average standard deviation of split frequencies < 0.05. Two separate analyses were performed: (i) considering the complete UL55 ORF for sequences publicly available in GenBank (*n* = 223) for 2 × 10^5^ states, and (ii) considering the section of the UL55 ORF covering the genotyping fragment for the Japanese sequences in this study (*n* = 49) for 5 × 10^5^ states. These sites were mapped to determine regions within gB that may be functionally or structurally relevant due to evolutionary pressure.

#### 2.2.8 Statistical Analysis

All statistical analyses were conducted using GraphPad Prism (GraphPad Software, San Diego, CA, USA). The Chi‐square test or Fisher's exact test was used for comparison of gB genotype distributions as appropriate. The Kruskal–Wallis test followed by Dunn's multiple comparison test were used for comparison of virus copy numbers between gB genotypes. The Mann–Whitney test was used for comparison of virus copy numbers between gB genotype groups in blood and ocular samples. A two‐tailed *t*‐test was used to compare the relative expression levels of peptide–HLA II complexes in the HLA binding assays. A *p *< 0.05 was considered statistically significant for all comparisons.

## 3 Results

### 3.1 Distinct Distribution of HCMV gB Genotypes in Blood and Intraocular Fluids of CMV‐Associated Diseases

We first performed sequence analysis of the UL55 region surrounding the furin‐cleavage site in 25 blood samples from patients with CMV viremia and 24 ocular fluid samples from patients with CMV‐associated ocular diseases to assess the presence and variability of this region (Figure 1A). Based on the obtained sequences, we confirmed that the samples could be classified into gB genotypes 1 to 3, using previously established genotype‐specific sequences (Figure 1B) [19].

To expand gB genotyping and to identify potential mix infections, multiplex SSP‐PCR was performed on 62 samples including the 49 samples previously subjected to sequencing analysis. The cohort comprised 27 blood samples obtained from patients with CMV viremia and 35 ocular fluid samples from patients with CMV‐associated ocular diseases. These samples were derived from 59 patients, among whom three patients with CMV retinitis had both blood and ocular fluid samples. Genotyping revealed the presence of gB1, gB2, and gB3, whereas gB4 and gB5 were not detected. Notably, the genotype distribution differed significantly between blood and ocular fluid samples (Fisher's exact test, *p *= 0.008) (Figure 1C). In blood, gB2 was most prevalent (41%, 11/27), followed by gB1 (33%, 9/27) and gB3 (11%, 3/27). In contrast, ocular fluid samples showed a dominance of gB3 (43%, 15/35), followed by gB1 (37%, 13/35), with gB2 least frequent (17%, 6/35). Additionally, mixed infections with multiple gB genotypes tended to be more frequent in blood samples (15%, 4/27) compared to ocular fluids (3%, 1/35). Among the three cases in which gB genotypes were determined in paired samples, one patient exhibited mixed gB1/gB2 genotypes in blood but only the gB1 genotype in the ocular fluid. In another patient, both gB1 and gB3 were detected in blood, whereas only gB3 was identified in the ocular sample. The third case showed an unexpected pattern: gB1 was detected in the blood sample, while gB2 was identified in the ocular fluid.

When stratifying ocular fluids by disease subtype, differences in gB genotype distribution were not significant (*p *= 0.54) (Table 1). In CMV retinitis, both gB1 and gB3 were most frequent (5/14 each; 35.7%), followed by gB2 (3/14, 21.4%). In CMV anterior uveitis, gB1 was present in 7/16 (43.8%), gB3 in 6/16 (37.5%), and gB2 in 3/16 (18.8%). Among CRN cases, gB3 was dominant (4/5, 80%), with gB1 in 1/5 (20%) and no detection of gB2. Overall, gB2 was least frequent across ocular diseases, while gB1 and gB3 were commonly detected in ocular fluid from both immunocompromised and immunocompetent patients with CMV ocular infection. Here all patients with CMV retinitis were immunocompromised, whereas all patients with CMV anterior uveitis were immunocompetent and not receiving immunosuppressive therapy, and that patients with CRN represented an intermediate immune status. As gB1 and gB3 dominated in ocular fluid regardless of immune status, we hypothesize that HCMV with these genotypes have likely adapted to the ocular environment and virus strains with different gB genotypes can become endowed with distinct tissue tropisms. Specifically, the gB2 appears more associated with hematogenous dissemination, whereas gB3 displays a higher affinity for ocular tissues. In contrast, gB1 was detected at comparable frequencies in both blood and ocular fluids, indicating more generalized tissue distribution.

**Table 1:** Distributions of gB genotypes in blood and ocular fluid.

Disease	Genotypes				
	gB1	gB2	gB3	Mix infection	
				gB1 + gB3	gB1 + gB2
Blood samples *n* = 27					
Viremia	9 (33.0%)	11 (41.0%)	3 (11.0%)	1 (4.0%)	3 (11.0%)
Ocular samples *n* = 35^a^					
Retinitis	5 (35.7%)	3 (21.4%)	5 (35.7%)	1 (7.1%)	0 (0%)
Anterior Uveitis	7 (43.7%)	3 (18.8%)	6 (37.5%)	0 (0%)	0 (0%)
CRN	1 (20%)	0 (0%)	4 (80%)	0 (0%)	0 (0%)

^a^ No significant difference among ocular diseases (*p* = 0.54, Fisher's exact test).

Next we examined whether gB genotypes were associated with viral load. We found that viral loads were significantly higher in gB1‐ and gB3‐positive samples compared with gB2‐positive samples in our cohort (Table 2, *p* = 0.016), and similar pattern was observed in blood samples (Table 3, *p* = 0.03). A comparable trend was noted in patients with CMV anterior uveitis, although the difference did not reach statistical significance due to the limited number of gB2 cases (Table 4). In contrast, CMV retinitis showed uniformly high viral loads regardless of genotype (Table 4). Collectively, these findings imply that gB1 and gB3 variants may replicate more efficiently in both blood and ocular compartments, thereby increasing their potential to cause ocular infection. The absence of a detectable difference in CMV retinitis likely reflects the fact that this disease is often diagnosed at a more advanced stage, when viral loads are already markedly elevated across genotypes.

**Table 2:** Viral load by gB genotype in all patients.

Genotype	*n*	Viral load	
gB1	22	2.24 × 10^5^ (1.70 × 10^4^−6.60 × 10^6^)	*
gB2	17	8.94 × 10^3^ (3.92 × 10^3^–1.61 × 10^5^)	
gB3	18	9.53 × 10^5^ (2.01 × 10^4^–1.85 × 10^7^)	**

*Note:* Copies/mL. Data are median (IQR: 25th–75th percentile). Kruskal–Wallis test; *p* = 0.016; Dunn's post hoc; mix infection was excluded.

* gB1 versus gB2; *p* = 0.02.

** gB3 vs gB2; *p* = 0.01.

**Table 3:** Viral load by gB genotype in blood samples.

Genotype	*n*	Viral load
gB1 and/or gB3	13	1.78 × 10^4^ (9.59 × 10^3^–1.50 × 10^5^)
gB2	11	6.60 × 10^3^ (1.50 × 10^3^–1.43 × 10^4^)

*Note:* Copies/mL. Data are median (IQR: 25th–75th percentile). Mann–Whitney *U* test; *p *= 0.03. Mix infection with gB1 and gB2 was excluded.

**Table 4:** Viral load by gB genotype in ocular diseases.

Disease	Genotype	*n*	Viral load
Anterior uveitis	gB1 and/or gB3	13	2.04 × 10⁵ (3.12 × 10⁴–9.53 × 10⁵)
	gB2	3	8.94 × 10³ (5.49 × 10³–2.88 × 10⁵)
Retinitis	gB1 and/or gB3	10	1.39×10⁷ (4.73 × 10⁶–3.65 × 10⁷)
	gB2	3	1.54 × 10⁷ (2.92×10⁶–8.72×10⁷)

*Note:* Copies/mL. Data are median (IQR: 25th–75th percentile).

Finally, we examined genetic mutations encoding amino acid substitutions within or its adjacent regions of the UL55 furin‐cleavage site but found no variants specifically associated with any CMV‐related ocular disease group.

### 3.2 Virus Peptide Derived From the Ocular gB Types Exhibited Lower Binding to HLA II

Previous studies have shown that gB is a major target of neutralizing antibodies in CMV‐seropositive individuals and that various gB‐derived peptides elicit CD4⁺T cell responses via HLA II presentation—critical for effective antibody production [30]. However, it remains unclear whether epitope differences among gB genotypes influence CD4⁺T cell recognition or contribute to immune modulation. To address this, we investigated whether peptides derived from different gB genotypes exhibit differential binding to HLA II molecules. Among 21 previously identified gB‐derived peptides known to activate CD4⁺T cells, five peptides showed amino acid variations between gB genotypes (Table S1) [30]. We then evaluated the predicted binding affinities of gB‐derived peptide pairs to HLA II allotypes present in our cohort using NetMHCIIpan‐4.3 (Tables S1 and S2). Among the peptides analyzed, two peptide pairs gB_190–204_ and gB_483‐497_ demonstrated greater than two‐fold difference in predicted binding affinity (%Rank) between gB genotypes on several HLA II molecules (Table 5). Peptide gB_190–204_ (gB1gB3)(SRVI **A** GTVFVAYHRD) was identical between gB1 and gB3, but distinct from the corresponding gB2 sequence, gB_190–204_ (gB2) (SRVI **G** GTVFVAYHRD). Peptide gB_483‐497_ (gB1gB2) (QLQFTYDTLR **G** YINR), shared by gB1 and gB2, differed from its gB3 counterpart, peptide gB_483‐497_ (gB3) (QLQFTYDTLR **S** YINR). Notably, peptides gB_190–204_ (gB1gB3) and gB_190–204_ (gB2) showed 2.9‐ and 2.5‐fold differences in predicted %Rank values for HLA‐DPB1*04:02 and HLA‐DQB1*03:01, respectively. Similarly, gB_483‐497_ (gB1gB2) and gB_483‐497_ (gB3) exhibited a two‐fold difference in predicted binding to HLA‐DRB1*01:01. These HLA II allotypes ranked among the fourth to fifth most prevalent variants (6%–11%) in our patient cohort and exhibit comparable frequencies in the general Japanese population (http://www.allelefrequencies.net/) (Table S2). Frequencies of other *HLA II* alleles in our cohort were also similar to the general Japanese population. No gB‐derived peptide sequences differing among gB genotypes were predicted to bind the most frequent HLA class II allotypes present in our cohort and in the general population.

**Table 5:** Selected peptide pairs derived from HCMV gB used in the MHC class II binding assay.

	Peptide Sequence	gB type	HLA class II allotype	Binding prediction (%Rank)	Figure 2
gB_190–204_	SRVI**A**GTVFVAYHRD	gB1/gB3	DPB1*0402	0.24	A
gB_190–204_	SRVI**G**GTVFVAYHRD	gB2	DPB1*0402	0.69	
gB_190–204_	SRVI**A**GTVFVAYHRD	gB1/gB3	DQB1*0301	1.22	B
gB_190–204_	SRVI**G**GTVFVAYHRD	gB2	DQB1*0301	0.49	
gB_483‐497_	QLQFTYDTLR**G**YINR	gB1/gB2	DRB1*0101	4.09	C
gB_483‐497_	QLQFTYDTLR**S**YINR	gB3	DRB1*0101	8.25	

*Note:* Binding affinities were predicted using NetMHCIIpan‐4.3.

**Figure 2:**
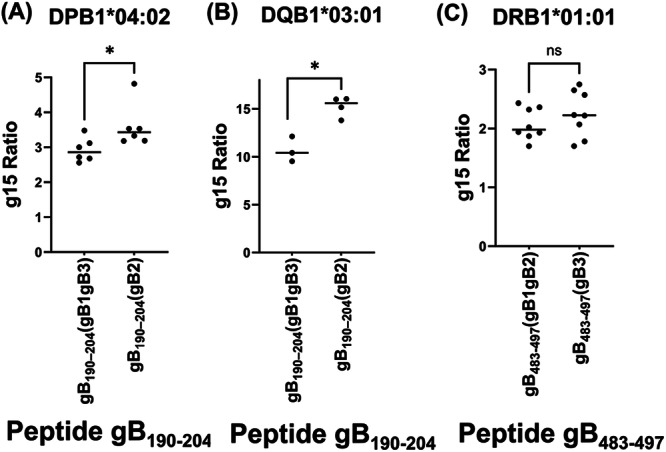
Virus peptide from glycoprotein B in the eye exhibited lower binding to HLA II. Cell surface expression of HLA–peptide complexes shown in Table 5 were evaluated using HLA II binding assay. (A) HLA expression was compared between peptides gB_190–204_ (gB1gB3) and gB2_(190–204)_ on HLA‐DPB1*04:02. (B) Comparison of peptides gB_190–204_ (gB1gB3) and gB2_(190–204)_ on HLA‐DQB1*03:01. (C) Comparison of peptides between gB_483‐497_ (gB1gB2) and gB_483‐497_(gB3) on HLA‐DRB1*01:01. Alpha chain alleles were DPA1*0103 for DPB1*0402, DQA1*05 for DQB1*03:01, and DRA*0101 for DRB1*01:01. *p < 0.05. two‐tailed *t*‐test.

To experimentally assess HLA II binding capacity, we performed an HLA expression assay that quantifies cell‐surface expression of the HLA‐peptide complex. We observed a significant reduction in the expression of HLA‐peptide gB_190–204_ (gB1gB3) complexes compared to HLA‐peptide gB_190–204_ (gB2) on both DPB1*04:02 and DQB1*03:01 (Figure 2A,B). Given that peptide gB190–204 (gB1/gB3), derived from the gB1 and gB3 genotypes enriched in ocular fluids, shows weaker HLA class II binding compared with the gB190–204 (gB2) peptide derived from the gB2 genotype which is predominant in blood, our findings suggest that ocular‐tropic gB variants may possess reduced HLA II binding capacity. This suboptimal presentation of gB1/gB3‐derived peptides could facilitate immune evasion by diminishing CD4⁺T‐cell recognition [38]. To confirm whether the gB190–204 (gB1/gB3) peptide sequence is actually present in ocular compartments, we verified the sequence by PCR and subsequent sequencing. In contrast, no significant difference in HLA‐peptide complex expression was observed between peptides gB_483‐497_ (gB1gB2) and gB_483‐497_ (gB3) (Figure 2C). Collectively, reduced antigen presentation of gB1/gB3 variants via HLA class II molecules and subsequent evasion of CD4⁺T‐cell immune surveillance may also facilitate viral expansion into the intraocular space.

### 3.3 K518R Substitution Around the Furin‐Cleavage Site Predominates in the Eye and in gB1 and gB3 Genotypes of Asian HCMV

**Figure 3:**
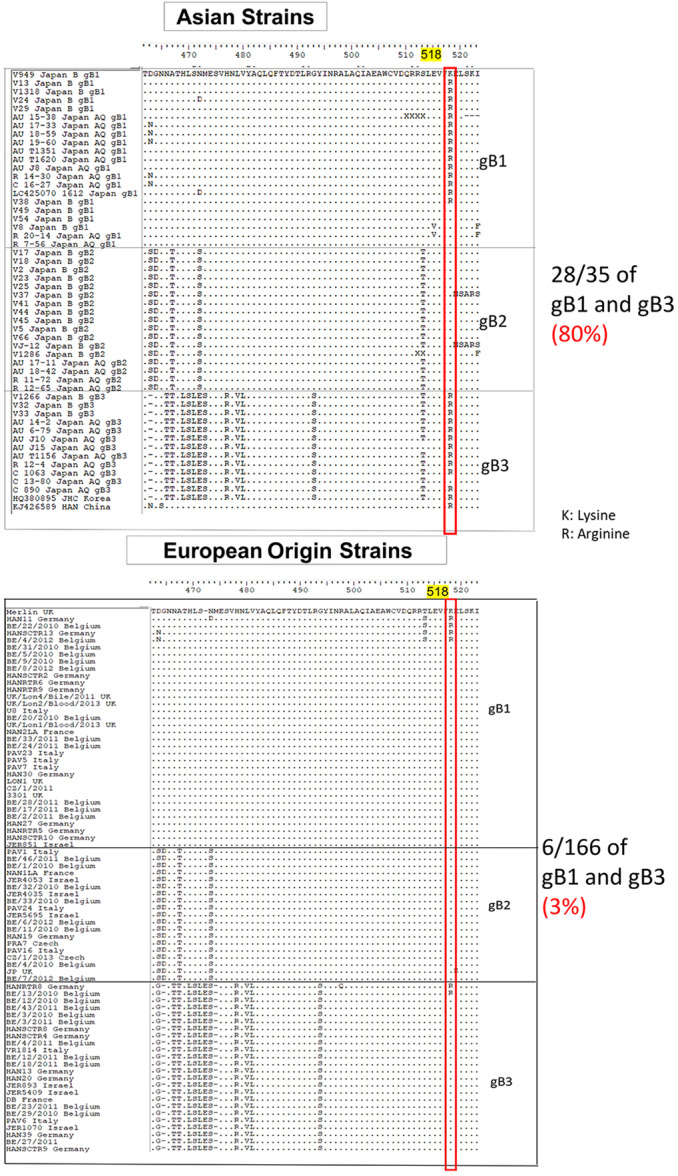
Predominance of the K518R substitution in gB1 and gB3 genotypes of HCMV from Asian populations. Amino acid sequences surrounding the furin cleavage site were compared among HCMV strains analyzed in this study, as well as sequences from public databases derived from Japan, China, South Korea, and 220 European origin strains. Among the 220 European origin‐derived strains, 166 were classified as gB1 or gB3. Sequences were grouped by gB genotype and geographic origin (Asian vs. European origin). The K518R substitution was detected in 80% (28/35) of Asian‐derived gB1/gB3 strains (including those from Japan, China, and South Korea), but in only 3% (6/166) of the corresponding European origin‐derived strains.

An outstanding question is whether Asian‐derived HCMVs have genetic characteristics distinct from European‐origin HCMV strains. Therefore, we compared the sequences of our samples with those in the GenBank database, including HCMVs from 220 European origin and three Asian strains (Table S3). The analysis identified a specific amino acid substitution, K518R, in most gB1 and gB3 of our samples and all Asian HCMV data (Figure 3). Notably, the substitution was not observed in gB2, the dominant type in blood samples. The K518R change was present in 28 out of 35 (80%) gB1 or gB3 of Asian HCMVs, but only in 6 of 166 (3%) European origin HCMVs with gB1 or gB3. Moreover, in our cohort, the substitution was detected in only 32% (8/25) of blood samples from patients with CMV viremia without CMV ocular involvement, whereas it was present in 71% (17/24) of the ocular fluid samples from patients with CMV ocular infection. This difference was statistically significant, showing a strong association of the substitution with ocular infections compared with viremia (Fisher's exact test, *p* = 0.01).

### 3.4 The K518R Substitution Is Predicted to Be Exposed on the Protein Surface and Increases the Local Molecular Stability in Domain III of gB

**Figure 4:**
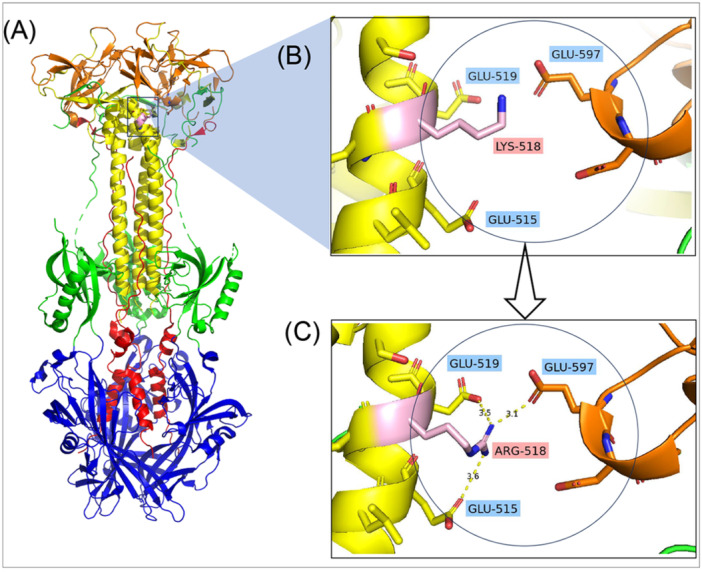
K518R substitution in HCMV glycoprotein B is surface‐exposed and enhances protein stability. The crystal structure of the extracellular domain of HCMV gB was visualized using PyMOL (PDB ID: 5CXF). Domains I–V are depicted in blue, green, yellow, orange, and red, respectively. (A) The position of the K518R substitution is indicated in pink and enclosed within a black box. (B) In the wild‐type structure, Lys518 does not engage in detectable interactions with surrounding residues. (C) In the mutant structure, substitution of lysine with arginine (K518R) introduces new electrostatic interactions with three acidic residues: Glu515 and Glu519 within Domain III, and Glu597 in Domain IV. These interactions are predicted to enhance local structural stability.

Given that most gB1 and gB3 genotypes—making up 83% of ocular fluids—harbor K518R, while less frequent gB2 lacked it, the substitution may influence gB binding to host cells in specific tissues. We next investigated how the K518R substitution might affect gB structure and function. To access the structural context of residue 518 in gB and predict potential binding interactions influenced by K518R, we conducted *in silico* modeling using PyMOL. The mutation was predicted to lie near the top of Domain III of gB (Figure 4). Structural modeling indicated that the K518R substitution enables new intradomain salt bridges with Glu515 and Glu519, and possibly an interdomain salt bridge with Glu597 in Domain IV, potentially stabilizing the Domain III‐IV interface in the postfusion conformation of gB [16].

### 3.5 The Furin Cleavage Region, Including K518R Substitution in the HCMV gB, Exhibits Evidence of Being Under Positive Selection

**Figure 5:**
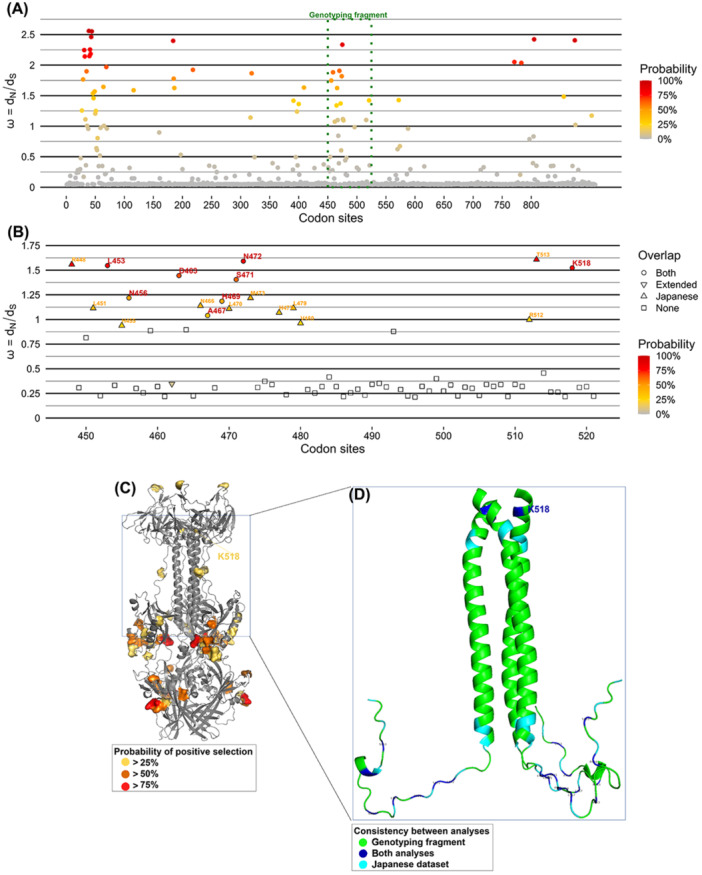
Bayesian selection analysis over the UL55. (A) Bayesian estimation of ω = dN/dS across the codons of UL55 for the extended dataset of sequence (*n* = 223). Horizontal and vertical axes represent the aligned codon sites and the corresponding ω, respectively. Dots are colored according to the inferred probability of being under positive selection, according to the legend on the right, between 25% (yellow) and 100% (red). The gene section corresponding to the amplified fragment in Japanese samples is highlighted whith a green box. (B) Bayesian estimation of *ω* = dN/dS across the codons of UL55 in the genotyping fragment for the Japanese dataset (*n* = 49). Horizontal and vertical axes represent the aligned codon sites and the corresponding ω, respectively. Dots are colored according to the inferred probability of being under positive selection, according to the legend on the right, between 25% (yellow) and 100% (red). Shapes follow the legend on the right, showing codon sites detected as under positive selection in both analyses (disc), only in the extended dataset (downward triangle), only in the Japanese dataset (upward triangle), or none (square). (C) Projection of results for the whole protein (A) into the predicted structure of UL55. The corresponding amino acid sites are colored by probability to be under positive selection. (D) Projection of results for the genotyping fragment (B) into the predicted structure of UL55. The amino acids are colored according to their detection in both analyses.

Assessing selective pressure on non‐synonymous substitutions is critical for understanding their functional and biological significance. Such analysis helps to distinguish whether an observed substitution enhances virus fitness, thereby increasing in frequency due to positive selection, or whether it is a result of genetic drift. To this end, we asked whether the K518R substitution has been subjected to positive selection through two Bayesian analyses to estimate selective pressures across the codon sites of gB from public databases, and the fragment encompassing the furin cleavage site with the Japanese sequences of this study. Remarkably, the region surrounding the furin cleavage site, including the K518R substitution, displayed signatureof positive selection in both analyses, inferring that the amino acid change in this region likely confers a selective advantage (Figure 5A,B). Moreover, other amino acid residues in gB with probabilities (≥ 25%) of positive selection were predominantly located on the extracellular domains (Figure 5C). It is noteworthy that despite the different composition of the datasets, both analyses coincided on the evidence supporting positive selection for multiple sites, therefore suggesting a persistent selective pressure over this region in HCMV (Figure 5D).

## 4 Discussion

To the best of our knowledge, this study is the first to compare the distribution of HCMV gB genotypes across different tissue compartments within the same population, combined with functional characterization of genotype‐specific features. Our analyses revealed a tissue‐specific distribution of gB genotypes, where gB3 was significantly enriched in ocular fluids from CMV‐associated ocular diseases, whereas gB2 was predominant in blood samples from patients with CMV viremia. gB1 was detected at comparable frequencies in both compartments. The viral loads were higher in gB1‐ and gB3‐positive samples compared with in those with gB2. Notably, gB1 and gB3 together accounted for 83% of ocular fluid samples, and a peptide shared between gB1 and gB3—distinct from that of gB2—exhibited reduced presentation by HLA II compared to the gB2‐derived peptide. Additionally, we identified a K518R substitution predominantly found in gB1 and gB3 genotypes of Asian HCMVs. The substitution was more frequently detected in the ocular fluids from patients with CMV ocular infection (71%), compared to blood from patients with CMV viremia (32%). Structural modelling suggested that this substitution may influence the conformation of gB protein, and signatures of positive selection on the substitution indicates potential functional and evolutionary significance, particularly in Asian HCMV.

A major finding of this study is the potential association between specific gB genotypes and their representation in distinct anatomical compartments. Previous studies of gB genotype distribution have largely focused on blood samples from immunocompromised individuals—including HIV‐positive patients, transplant recipients, and congenitally infected neonates—where gB1 or gB2 have often been reported as the predominant genotypes, although the dominant type has varied across studies [39, 40, 41]. Therefore, it has remained unclear whether genotype distributions observed in a single compartment reflect tissue tropism or regional virus prevalence. Earlier studies that compared multiple components used techniques with lower resolution, such as restriction fragment length polymorphism analysis, and yielded inconsistent results in blood and ocular fluids [28, 42, 43, 44].

Although genotypic differences in gB have been implicated in viral tropism and immune evasion, the functional consequences remain poorly understood. In our study, viral loads were significantly higher in gB1‐ and gB3‐positive samples than those in harboring gB2, implying that HCMV with gB1 and gB3 genotypes may possess greater replicative capacity. Such enhanced replication could, in turn, facilitate viral dissemination from the bloodstream to ocular tissues. Furthermore, the peptide spanning residues 190–204 which is conserved in gB1 and gB3 but distinct in gB2, exhibited reduced HLA II presentation, potentially diminishing CD4⁺T cell recognition. Such impaired antigen presentation may facilitate immune escape of HCMV with gB1/gB3 and promote efficient dissemination to immune‐privileged intraocular sites. Recent reports from China and Taiwan corroborate the potentially pathogenic role of the gB3 variant suggested by our findings. In these studies, gB3 was linked to more severe or bilateral manifestations of CMV anterior uveitis, reinforcing its possible involvement in ocular pathogenesis [26, 45]. Minor discrepancies were noted between the predicted and measured binding hierarchy for DPB1*04:02; however, since both peptides fell within the strong‐binding range, this difference is unlikely to be biologically meaningful.

**Figure 6:**
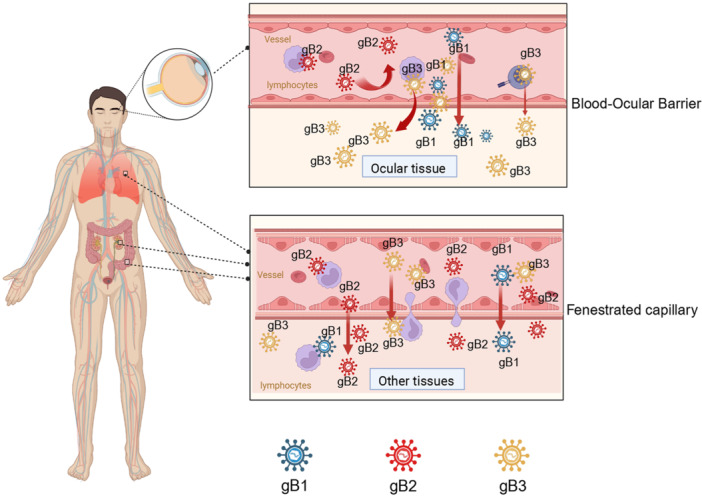
Potential roles of the blood‐ocular barrier in restricting HCMV entry into ocular tissues in a type‐specific manner. HCMV latently infected in blood leukocytes circulates systemically. Upon reactivation, HCMV can disseminate into peripheral organs, including the lungs and the eye. The distinct distribution of HCMV types between the blood and ocular tissues suggests that the blood‐ocular barrier may limit HCMV spread beyond the blood endothelium, preventing virus expansion into the intraocular space. Only specific virus types may be able to cross this barrier.

Our findings of tissue‐specific viral genotypes align with previous studies showing distinct UL40 signal peptide variants in ocular fluids compared to blood, but not in other compartments such as the lung [46, 47]. Moreover, a recent study analyzing viral genomes across tissues from the same individuals reported substantial variation only in ocular fluids, suggesting the eye as a site of viral adaptation [48]. While HCMV can establish latency in circulating leukocytes and cross fenestrated endothelium in many organs, the blood–ocular barrier may selectively filter virus with distinct genotypes, favoring those with enhanced entry capacity and/or immune evasion at entrance of the eye (Figure 6).

This study also provides the first characterization of gB molecular features in Asian HCMVs compared to those from individuals of European origin. With CMV seroprevalence exceeding 80%–90% and a high incidence of CMV anterior uveitis in Asia, characterizing regional viral genetic diversity is of prime importance [6, 7, 49]. Our analysis identified the K518R substitution as prevalent in Asian HCMVs carrying gB1 and gB3, but rare in HCMV of European origin. Structural modeling implied that this substitution, located near the top of the gB core within Domain III, enhances local molecular stability by promoting intra‐ and interdomain salt bridge formation, particularly at the interface with Domain IV. As a class III viral fusion protein, HCMV gB is structurally homologous to herpes simplex virus 1 gB, where Domains III and IV form a central structural core in the protein. Mutations or insertions in these regions have been shown to severely impair cell surface expression [50, 51]. A recent mutagenesis study of HCMV gB further demonstrated that single‐point mutations in Domain III disrupt virus entry and replication, likely by disrupting membrane fusion during virus entry [52]. Although the K518R mutation has not yet been functionally characterized, its location at a critical structural interface and evidence of positive selection suggest that it influences gB stability, potentially affecting cellular entry efficiency and cell‐to‐cell spread. Of note, a recent report compared the infectivity between HCMV strains with different gB variants isolated from Chinese patients by swapping the gB gene variants into a TB40/E backbone [53]. In this study, two gB variants carrying the K518R substitution exhibited higher infectivity, whereas two variants lacking the K518R showed lower infectivity. Based on these findings, we hypothesize that the K518R substitution may enhance virus infectivity and contributes to the higher prevalence of CMV‐associated ocular diseases in Asia by promoting viral fitness within the ocular niche.

gB remains a major antigenic target in CMV vaccine development. A vaccine could serve as a more potential alternative to current therapies that require prolonged antiviral therapies for CMV‐associated ocular diseases. Among the promising candidates, the gB/MF59 vaccine—based on gB1 sequence from the Towne strain—has shown protection against gB1 and gB2 strains but not against gB3, the most frequent genotype found in the eye [54]. Our findings emphasize the importance of considering gB genotypes in the design of next‐generation CMV vaccines.

While our study reveals genotype differences between blood and ocular fluids, we did not observe significant variation in gB region among different types of CMV‐associated ocular diseases. We acknowledge limitations in functionally confirming the impact of specific substitutions on protein structure and tissue affinity. Sequence variability in gB regions outside the furin cleavage site, particularly within the N‐ and C‐terminal domains, may modulate virus–host interactions [12]. Limited variation in the N‐terminal surface‐exposed region could subtly influence antibody recognition, while C‐terminal cytoplasmic polymorphisms may influence immune responses rather than directly determining tissue tropism [12, 55]. Furthermore, viral entry and membrane fusion rely on coordinated interactions among multiple envelope glycoproteins, including the gH/gL complex, which acts together with gB to facilitate membrane fusion [56]. Therefore, the contribution of other glycoproteins to viral infectivity and ocular dissemination should also be considered [12]. Further experimental validation is required to clarify these mechanisms. Continued research into the genetic diversity and functional consequences of HCMV envelop proteins will be essential for understanding disease pathogenesis and guiding improved treatment strategies.

## Author Contributions

Tantri Lestari conceived and designed this study, executed experiments, conducted analysis and interpretation of data, and drafted the manuscript. Nobuyo Yawata conceived and designed the study, supervised and administered the projects, writing and performed critical revision of the manuscript, and obtained funding. Gabriel Gonzalez contributed to genomic data analysis, protein and positive selection analysis, and writing and critical review of the draft. Hiroko Miyadera designed and conducted HLA II expression analysis, drafted and critically reviewed the manuscript. Daisuke Motooka, Yuko Imamura, and Hiroya Oki contributed to genetic and protein analysis and critical review of the manuscript. Yasuo Mori contributed to patient recruitment and sample collection. Mariko Shirane contributed methodology and sample collection. Seik‐Soon Khor, Mihoko Shimada and Yosuke Omae, Katsushi Tokunaga conducted HLA analysis. Dyah Ayu Windy, Chihiro Fukui, Hiroki Tsutsui, Shiori Kuramoto, Riku Nakamura, Satoshi Yamana, Toshikatsu Kaburaki, Hisashi Mashimo, Hiroshi Takase, Ryoji Yanai, Eiichi Hasegawa, Nobuyuki Ohguro contributed to sample collection, clinical assessment, and critical revision of the manuscript. Satoko Nakano contributed methodology and critical revision of the manuscript. Kensuke Shibata and Makoto Yawata contributed interpretation of data and critical revision of the manuscript. Koh‐Hei Sonoda contributed to supervision, resources, funding acquisition, and critical revision of the manuscript.

## Ethics Statement

This study was conducted in accordance with the Declaration of Helsinki and was approved by the Institutional Review Board of Kyushu University Hospital (No. 21159, M23009). Written informed consent was received from all participants.

## Conflicts of Interest

The authors declare no conflicts of interest.

## Supporting information


**Table S1.** Peptide candidates for HLA peptide binding Assay. **Table S2.** *HLA II* allele frequencies in this study. **Table S3.** Accession numbers of CMV whole genome sequences of European origin strains and Asian strains.

Supplementary Tables 251205.

## Data Availability

The data that support the findings of this study are available from the corresponding author upon reasonable request.
