# Supplementary material for: Molecular Variations in Glycoprotein B of Asian Human Cytomegalovirus: Potential Impact on Virus Entry and Immune Evasion in Ocular Diseases
Source: J Med Virol. 2026 Jan 7;98(1):e70786. doi: 10.1002/jmv.70786 (PMC12778896; doi:10.1002/jmv.70786)
Supplement: Supplementary file 1 — Table S1. Peptide candidates for HLA peptide binding Assay. Table S2. HLA II allele frequencies in this study. Table S3. Accession numbers of CMV whole genome sequences of European origin strains and Asian strains. [file JMV-98-e70786-s002.docx]

Supplementary Table Legends

**Table S1. Peptide candidates for HLA peptide binding Assay**. Five peptides were selected that demonstrated variations by gB genotypes from 21 immunogenic UL55 peptides (30). The type of gB genotype associated with each peptide sequence is indicated in the represented column. The binding prediction (%Rank) using the NetMHCIIpan-4.3, are shown. The %Rank below 1 indicates strong binding, between 1 and 5 indicates weak binding, and above 5 indicates no binding.

**Table S2. *HLA II* allele frequencies in this study**. The frequencies of individual alleles of *HLA II DR*, *DQ*, and *DP* were examined within the patient cohort. The *HLA II* alleles DRB*01:01, DQB*03:01, and DPB*04:02 tested in the MHC-peptide binding assay are shown in red and bold.

**Table S3 Accession numbers of CMV whole genome sequences of European origin strains and Asian strains.** The NCBI accession numbers for the 220 European ancestry strains, 1 Japanese, 1 Korean, and 1 China strain employed in this analysis were compiled.
